# Generalizability of anti–SARS-CoV-2 seroprevalence estimates to the Montréal pediatric population: a comparison between 2 weighting methods

**DOI:** 10.1093/aje/kwae276

**Published:** 2024-08-12

**Authors:** Adrien Saucier, Bouchra Nasri, Britt McKinnon, Mabel Carabali, Laura Pierce, Katia Charland, Kate Zinszer

**Affiliations:** Université de Montréal Centre de recherche en santé publique, Montréal, Quebec, Canada; Université de Montréal Centre de recherche en santé publique, Montréal, Quebec, Canada; Université de Montréal Centre de recherche en santé publique, Montréal, Quebec, Canada; Université de Montréal Centre de recherche en santé publique, Montréal, Quebec, Canada; Université de Montréal Centre de recherche en santé publique, Montréal, Quebec, Canada; Université de Montréal Centre de recherche en santé publique, Montréal, Quebec, Canada; Université de Montréal Centre de recherche en santé publique, Montréal, Quebec, Canada

**Keywords:** COVID-19, SARS-CoV-2, seroprevalence, weighting methods

## Abstract

Seroprevalence studies of SARS-CoV-2 infections often have been based on study populations with nonrandom and nonrepresentative samples, limiting the generalizability of their results. In this study, the representativity and the generalizability of the baseline estimate (data collected from October 16, 2020, to April 18, 2021) of a pediatric seroprevalence study based in Montréal were investigated. The change in the estimates of seroprevalence were compared between 2 different weighting methods: marginal standardization and raking. The target population was the general pediatric population of Montréal, based on 2016 Canadian census data. Study results show variation across the multiple weighting scenarios. Although both weighting methods performed similarly, each possesses its own strengths and weaknesses. However, raking was preferred for its capacity to simultaneously weight for multiple underrepresented study population characteristics.

## Introduction

Seroprevalence studies were needed throughout the COVID-19 pandemic to estimate and monitor the prevalence and distribution of SARS-CoV-2 infections. SARS-CoV-2 infection testing resources were often limited to priority populations (eg, health care workers, senior citizens) or symptomatic individuals and varied by setting and over time, which led to a considerable portion of infections not being reported or captured by surveillance systems.^1^^,^^2^ Undetected infections were particularly common in pediatric populations because SARS-CoV-2 infections in children are often asymptomatic.^3-5^ Hence, multiple seroprevalence studies have been conducted among pediatric populations to estimate the prevalence of SARS-CoV-2 infection through assessments of immunoglobulin G antibody response to infection.^6^

Given the emergency context, particularly in the beginning phases of the pandemic, seroprevalence studies were often conducted using nonrandom convenience sampling.^7-14^ This sampling approach can lead to various methodological challenges, including lack of representativeness.^15^ For descriptive studies aiming to measure the occurrence of SARS-CoV-2 infections in a broader target population, nonrepresentative samples can limit the application of results outside of the study population.^16-18^ The direct application of an estimate based on a nonrandom sample to a target population can lead to under- or overestimation of the disease occurrence and misidentification of high-risk groups.^17^ When external data on the target population are available, descriptive studies should consider characterizing study population deviation from the target population and apply appropriate standardization methods to improve the generalizability of their results.^19^ Weighting is a key tool to reduce the effect of sample nonrepresentativeness.^20^ Weighting has been used in some SARS-CoV-2 seroprevalence studies, although few studies provide the details needed to fully understand how the weighting was conducted.^9^^,^^21^ In addition, weighting is often only applied to 1 characteristic (eg, ethnicity), which can limit the representativity of the estimation on other study population characteristics.^9^^,^^21^

Marginal standardization and raking are methods that can be used to standardize estimates to a target population. Marginal standardization estimates the prevalence in a target population from a regression analysis of sample data by creating weights based on the regression coefficients and the explanatory variable distributions of the target population.^22^ Raking is an iterative method that applies a proportional adjustment to the sample characteristics to match the corresponding characteristics’ marginal totals in the target population.^23^ Raking has been used to standardize SARS-CoV-2 seroprevalence according to the distribution of multiple characteristics in a target population.^24-26^ In this study, using data from the *Enfants et COVID-19: Étude de séroprévalence* (EnCORE) cohort study baseline assessment (October 2020 to April 2021), we compare the application of marginal standardization and raking to estimate the seroprevalence of SARS-CoV-2 infection in the target population of children aged 2-17 years in Montréal, Canada.

## Methods

### Data and survey description

**Figure 1:**
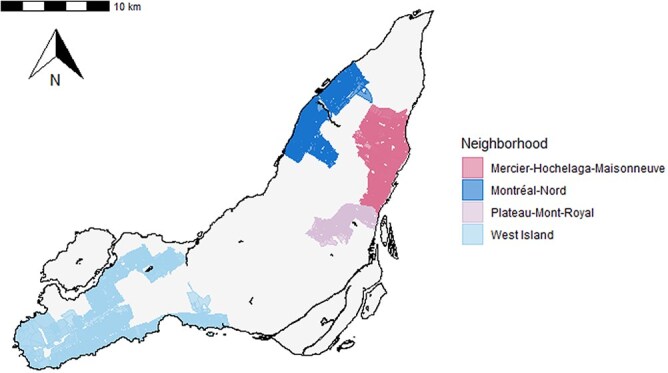
Neighborhoods with participating schools and daycares, *Enfants et COVID-19: Étude de séroprévalence* (EnCORE) study, Montréal, Québec, Canada, 2020-2021.

This study reanalyzes baseline data from the *Enfants et COVID-19* (EnCORE) study,^27^^,^^28^ a cohort study with the objective to estimate seroprevalence to anti–SARS-CoV-2 antibodies among study participants aged 2-17 years from October 2020 to June 2023. Children were recruited from 4 neighborhoods of Montréal (Figure 1) to reflect variability in socioeconomic status based on the *indice de milieu socioéconomique*, a socioeconomic index used by Québec Ministry of Education.^27^ Recruitment began in the West Island neighborhood at the end of October 2020 and in Plateau-Mont-Royal in mid-November 2020 and was then extended to Mercier-Hochelaga-Maisonneuve and Montréal-Nord at the end of November 2020. The initial aim of the study was to estimate seroprevalence in the 4 neighborhoods according to age groups (2-4, 5-9, 10-14, and 15-17 years) with a 2% precision, given a confidence level of .95 and assuming loss to follow-up of 30%.^27^

Recruitment followed 2 stages: (1) public school boards and daycare administrations located in the 4 neighborhoods were invited to participate in the study; and (2) invitation emails were sent by participating schools and daycare administrations to parents of all eligible students. Dried blood spot specimens were collected at home by participants and sent to a laboratory for serological analyses conducted using enzyme-linked immunosorbent assays, with a 95% sensitivity and 100% specificity using the receptor-biding domain from the spike protein of SARS-CoV-2.^27^ Baseline data were collected from October 16, 2020, to April 18, 2021 and there were 4 subsequent rounds of data collection. Although EnCORE is a longitudinal study, the present study compares different weighting methods to analyze cross-sectional data that were collected at baseline. Further details of participant recruitment and data collection have been described previously.^27^^,^^28^

Missing values were assessed in the study population, and multiple imputation by chained equations (MICE) was conducted to generate 20 imputed data sets, which were pooled using Rubin’s rule.^29^^,^^30^^(chapter 2)^ More key details about the imputation procedure can be found in Figures S1 and S2, and Tables S1 and S2. Data were missing across several variables, with the greatest amount of missingness occurring for household income (31.6%) and parent’s birthplace (31.9%). These variables were collected only at the first follow-up (May-September 2021) and, hence, were exposed to loss to follow-up between the baseline and the follow-up. All other characteristics had less than 3% of missingness. Every variable that presented some part of missingness was imputed. Stratified tables were created to investigate if any differential patterns existed between missingness in household income or parent’s place of birth and seropositivity.

### Statistical analysis

Univariate descriptive statistics were computed for our study population, and crude seroprevalence estimates were calculated by sociodemographic characteristics using bivariate stratified proportions with 95% CIs. To assess the representativity of the EnCORE sample across participant and household characteristics, we compared descriptive univariate statistics for 8 key characteristics from the sample to the target population through absolute differences in percentage and χ^2^ tests. The target population was the population of children ages 2-17 years old living in 1 of the Island of Montréal forward sortation areas (FSAs). An FSA is a geographic unit based on the first 3 characters in the Canadian postal code.^31^ The Island of Montréal is 499 km^2^ with 1 942 044 residents in 2016 and contains 107 FSAs.^32^ Finally, we applied marginal standardization and raking to weight the seroprevalence estimate so it would be representative of our target population.

### Marginal standardization

Marginal standardization uses statistical modeling and allows the weighting for a particular covariate based on its “marginal effect,” which is estimated by a regression coefficient.^22^ For each weighting scenario, the weighted covariate distribution was applied to the study population, and the other covariates in the model were set to their observed values in the data set. In our study, weights were defined as the proportion of individuals with a given characteristic in the target population, based on census data. Of 8 sociodemographic variables identified as key characteristics, we identified 7 variables that were under- or over-represented in the study population for weighting in marginal standardization. To guide the variable selection of our marginal standardization models,^22^ we developed a directed acyclic graph (DAG) based on the scientific literature, including DAGs from 2 research articles.^33^^,^^34^ Variable selection for logistic models regressing seropositivity on different covariates was guided by our DAG (Figure S3).^16^^,^^22^^,^^34^^,^^35^ For each of the 7 under- or over-represented key characteristics, we found the minimal adjustment set of hypothesized confounders and built logistic regressions model (Appendix S2, Table S3). Because marginal standardization operates with regression coefficients, a minimally sufficient adjustment set is needed for each variable that will be weighted to generate new predicted probabilities of the outcome with the least confounded association possible (Appendix S2, Table S4). The positivity assumption was verified for each weighting model via contingency tables, and the presence of influential points and the presence of clustering (Appendix S2, Tables S5 and Table S6) were systematically assessed.

### Raking

We considered the same 7 characteristics that were under- or over-represented in the study population for raking weights. Raking only requires the target population marginal totals of the characteristics that will be weighted, because it iteratively calculates the proportional weighting for each of the specified variables until the sample distribution reaches the target population distribution.^23^^,^^36^ To simultaneously weight multiple variables, the process is carried out until there is a balance in the distribution between all the sample characteristics and the target population.^23^ The precision of the estimates generated by raking and marginal standardization methods is further compared in Appendix S3.

### Weighted characteristics

Sociodemographic and socioeconomic variables that were identified to be weighted were selected based on the literature and also needed to be available for both the study population and the target population (via Canadian census data). The EnCORE online survey collected the following sociodemographic variables, which were categorized for these analyses as age (2-4, 5-9, 10-14,and 15-17 years), sex assigned at birth (male or female), parent’s ethnic/racial minority status (yes or no), parent’s level of education (less than a bachelor degree, bachelor degree, and master degree or more), presence of an essential worker in the household (essential worker in health sector, essential worker not in the health sector, no essential worker), number of bedrooms, parent’s place of birth (Canada or outside Canada), and household income before taxes (CAD$ <100,000 year^−1^ or ≥$100 000 year^−1^). Parent’s place of birth and household income were not collected at baseline, both were first collected at the second point of data collection (May 2021 to September 2021). Essential workers included the following occupations^37^: health care worker, day care educator or worker, corrections or prison officer, teacher or other school staff, first responder, public transportation driver, food service industry, grocery store staff, pharmacy staff, hairdresser or barber, aesthetician, flight attendant and factory worker. Household density was computed as the ratio of persons per bedroom. Ethnic/racial minority status is based on Statistics Canada definition of visible minority: “individuals, other than Aboriginal peoples, who are non-Caucasian in race or non-white in skin color.”^38^

Canadian census data (2016)^39^ were used to compare the distribution of sociodemographic and socioeconomic characteristics in the study population with that of the general pediatric population (2-17 years old) on the Island of Montréal. Census weight proportions were computed for the target population by restricting to households in FSAs of the Island of Montréal containing at least 1 child from 2 to 17 years old which corresponded to a pediatric population of approximately 313 505 persons.

We present additional analyses based on complete-case data and imputed data to detect any major difference in coefficient estimation. All analyses were carried out with R (version 4.1.2) using principally the *MICE*, *marginaleffects*, and *survey* libraries. ^40-42^

## Results

There were 1632 children who submitted a serological specimen and completed an online survey for the baseline collection. The study population’s mean age was 9 years old and mostly composed of children living in the West Island area (30.9%) or the Plateau-Mont-Royal neighborhood (32.7%). Most participants provided a dried blood spot specimen date-stamped between December 2020 to January 2021 (47.5%) (Table 1). Stratified tables showed that missingness for household income and parent’s place of birth tended to be higher among seronegative participants. Crude seroprevalence was higher in Montréal-Nord (9.3%), in children with a parent from an ethnic/racial minority background (10.7%) and among children from a household with an annual income of less than CAD$100 000 (8.2%) (Table 2).

**Table 1:** Baseline characteristics of participating children, *Enfants et COVID-19: Étude de séroprévalence* (EnCORE) study, 2020-2021.

**Variable**	**Frequency, no. (%)**		
	**Study population (*n* = 1632)**	**Complete case sample (%)**	**Imputed sample, (n = 1632)**
Timing of dried blood spot specimen collection			
October-November 2020	377 (23.1)	201 (20.3)	377 (23.1)
December-January 2021	776 (47.5)	497 (50.3)	776 (47.5)
February-April 2021	479 (29.4)	290 (29.4)	479 (29.4)
Biological sex at birth			
Female	801 (49.1)	481 (48.7)	801 (49.1)
Male	831 (50.9)	507 (51.3)	831 (50.9)
Age, years			
2-4	333 (20.4)	229 (23.2)	333 (20.4)
5-9	543 (33.2)	340 (34.4)	543 (33.2)
10-14	531 (32.6)	298 (30.2)	531 (32.6)
15-17	225 (13.8)	121 (12.2)	225 (13.8)
Neighborhood			
West Island	504 (30.9)	272 (27.5)	504 (30.9)
Plateau-Mont-Royal	534 (32.7)	358 (36.2)	534 (32.7)
Mercier-Hochelaga Maisonneuve	357 (21.9)	239 (24.2)	357 (21.9)
Montréal-Nord	237 (14.5)	119 (12.1)	237 (14.5)
Household density, individuals per bedroom			
<2	1370 (83.9)	837 (84.8)	1407 (86.2)
≥2	221 (13.5)	151 (15.3)	225 (22.8)
Missing data	41 (2.5)		
Parent’s ethnic/racial minority status			
Racial/ethnic minority	201 (12.3)	125 (12.7)	206 (12.6)
Not a racial/ethnic minority	1406 (86.2)	863 (87.3)	1426 (87.4)
Missing data	25 (1.5)		
Parent respondent level of education			
Less than bachelor degree	384 (23.5)	182 (18.4)	389 (23.8)
Bachelor degree	647 (39.6)	419 (42.4)	655 (40.1)
Master degree or more	581 (35.6)	387 (39.2)	588 (36.0)
Missing data	20 (1.2)		
Household income before taxes, CAD$			
<100 000	328 (20.1)	320 (32.4)	564 (34.6)
>100 000	686 (42.0)	668 (67.6)	1068 (65.4)
Missing data	618 (37.9)		
Essential worker in the household			
No essential worker in the household	913 (55.9)	548 (55.5)	920 (56.4)
≥1 essential worker, health domain	267 (16.4)	182 (18.4)	269 (16.5)
≥1 essential worker, not health	441 (27.0)	258 (26.1)	443 (27.1)
Missing data	11 (0.7)		
Parent respondent place of birth			
Canada	851 (52.1)	761 (77.0)	1244 (76.2)
Outside Canada	261 (16)	227 (23.0)	388 (23.8)
Missing data	520 (31.9)		

**Table 2:** Seropositivity per imputed characteristics of participating children, *Enfants et COVID-19: Étude de séroprévalence* (EnCORE) study, 2020-2021.

	**Seropositive, no./total**	**%**	**95% CI**
Total	95/1632	5.8	4.7-7.0
Timing of dried blood spot specimen collection			
October-November 2020	11/377	2.9	1.2-4.6
December-January 2021	38/776	4.9	3.4-6.4
February-April 2021	46/479	9.6	7.0-12.2
Sex at birth			
Female	55/801	6.9	5.1-8.6
Male	40/831	4.8	3.4-6.3
Age, years			
2-4	16/333	4.8	2.5-7.1
5-9	27/543	5.0	3.1-6.8
10-14	36/531	6.8	4.6-8.9
15-17	16/225	7.1	3.8-10.5
Neighborhood			
West Island	17/504	3.4	1.8-4.9
Plateau-Mont-Royal	28/534	5.2	3.4-7.1
Mercier-Hochelaga-Maisonneuve	28/357	7.8	5.0-10.6
Montréal-Nord	22/237	9.3	5.6-13.0
Household density, individuals per bedroom			
<2	78/1407	5.5	4.3-6.7
≥2	17/225	7.6	6.2-8.8
Parent’s racial/ethnic minority status			
Racial/ethnic minority	22/206	10.7	6.5-14.9
Not a racial/ethnic minority	73/1426	5.1	4.0-6.3
Parent’s level of education			
Less than bachelor degree	18/389	4.6	2.5-6.7
Bachelor degree	41/655	6.2	4.4-8.1
Master degree or more	36/588	6.1	4.2-8.1
Household income, CAD$			
100 000	46/564	8.2	6.0-10.5
>100 000	49/1068	4.5	3.3-5.8
Essential worker in household			
No essential worker	44/920	4.8	3.4-6.2
≥1 essential worker, health domain	21/269	7.8	4.6-11.0
≥1 essential worker, not health	30/443	6.8	4.4-9.1
Parent’s place of birth			
In Canada	64/1244	5.2	3.9-6.4
Outside Canada	31/388	7.9	5.2-10.5

We compared the covariate distributions of our sample and Montréal’s overall pediatric population (Table 3), which showed that in comparison to the pediatric population living in any FSA on the Island of Montréal, the *EnCORE* sample had 30.4% fewer children with a parent from an ethnic/racial minority background, 32.8% fewer children with a parent having less than a bachelor’s degree and 32.5% fewer children from a household with an annual income of less than CAD$100 000.

**Table 3:** Relative frequencies comparison between imputed sample population and general population for all forward sortation areas on the Island of Montréal.

	**Census, % (95% CI)**	**Study population, % (95% CI)**	**Difference (95% CI)**	**χ** ^**2**^ **(*P* value)**
Female sex at birth	49.0 (48.8-49.2)	49.1 (46.6-51.5)	0.1 (−2.5 to 2.4)	0 (1.00)
Age, years				
2-4	20.9 (20.8-21.1)	20.4 (18.5-22.5)	0.05 (−1.5 to 2.5)	0.25 (.62)
5-9	33.3 (33.1-33.4)	33.3 (31.0-35.6)	0.0	0 (1.00)
10-14	28.7 (28.5-28.8)	32.5 (30.3-34.9)	−3.8 (−6.2 to −1.6)	11.67 (< 0.05)
15-17	17.1 (17.0-17.2)	13.8 (12.2-15.6)	3.3 (1.6-5.0)	12.54 (< .05)
Household density <2 persons per bedroom	72.1 (72.0-72.3)	86.2 (84.4-87.8)	−14.1 (−15.8 to −12.3)	159.49 (< .05)
Parent’s visible minority status: visible minority	43.1 (43.8-44.2)	12.6 (11.1-14.4)	30.4 (28.8-32.1)	614.7 (< .05)
Parent’s level of education				
Less than bachelor degree	56.6 (56.4-56.8)	23.8 (21.8-26.0)	32.8 (30.7-34.9)	708.21 (*<* .05)
Bachelor degree	24.2 (24.1-24.4)	40.1 37.8, 42.6	−16.0 (−18.3 to −13.5)	222.53 (< .05)
Master degree or more	19.2 (19.0-19.3)	36.0 (33.7-38.4)	16.9 (−19.2 to −14.5)	296.1 (< .05)
Household income CAD$ <100 000	67.0 (66.7-67.2)	34.5 (32.3-36.9)	32.5 (30.0-34.7)	762.42 (< .05)
Essential worker in household				
No essential worker	62.2 (62.1-62.4)	56.4 (53.9-58.8)	5.8 (3.4-8.3)	23.52 (< .05)
≥1 essential worker, health domain	11.7 (11.5-11.8)	16.5 (14.7-18.4)	−4.8 (−6.7 to −3.0)	36.19 (< .05)
≥1 essential worker, not health	26.1 (26.0-26.3)	27.2 (25.0-29.4)	1.0 (−3.2 to 1.2)	0.86 (.35)
Parent’s place of birth: Canada	41.7 (41.6-41.9)	76.2 (74.1-78.3)	−34.5 (−36.6 to 32.4)	791.73 (< .05)

**Figure 2:**
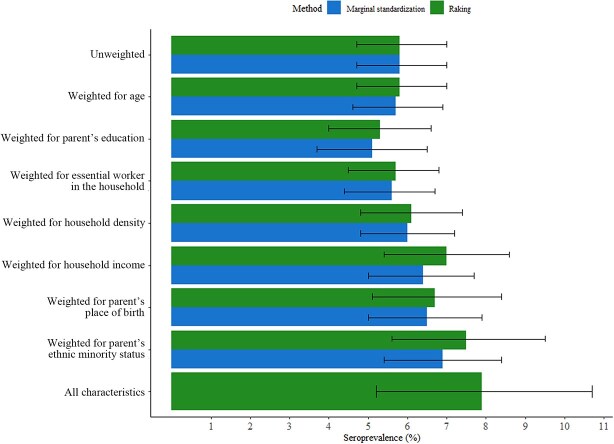
Seroprevalence estimates weighted by marginal standardization and raking, *Enfants et COVID-19: Étude de séroprévalence* (EnCORE) study, 2020-2021.

The unweighted seroprevalence estimate was 5.8% (95% CI, 4.7-7.0) in comparison with the weighted estimates, which varied between 5.1% (95% CI, 3.7-6.5) and 6.9% (95% CI, 5.4-8.4) with the marginal standardization procedure, depending on the weighted covariate (Table 4, Figure 2). Individual weighting by household income and parent’s ethnic/racial minority status, or place of birth increased the estimated seroprevalence by 0.6, 1.1, and 0.7 percentage points, respectively. Weighting for parent’s education decreased seroprevalence by 0.7 percentage points. Weighting for multiple covariates simultaneously was not possible with marginal standardization because no minimal adjustment set could be identified by our DAG that would allow us to simultaneously estimate the least confounded association for more than 1 weighting covariate.

**Table 4:** Comparison between unweighted and weighted seroprevalence estimates, *Enfants et COVID-19: Étude de séroprévalence* (EnCORE) study, 2020-2021.

	**Overall seroprevalence (95% CI)**	
	**Marginal standardization**	**Raking**
Unweighted	5.8 (4.7-7.0)^a^	5.8 (4.7-7.0)
Age	5.7 (4.6-6.9)^b^	5.8 (4.7-7.0)
Weighted for parent’s ethnic/racial minority status	6.9 (5.4-8.4)^b^	7.5 (5.6-9.5)
Weighted for household income	6.4 (5.0-7.7)^c^	7.0 (5.4-8.6)
Weighted for parent’s education	5.1 (3.7-6.5)^b^	5.3 (4.0-6.6)
Weighted for essential worker in the household	5.6 (4.4-6.7)^d^	5.7 (4.5-6.8)
Weighted for parent’s place of birth	6.5 (5.0-7.9)^b^	6.7 (5.1-8.4)
Weighted for household density	6.0 (4.8-7.2)^e^	6.1 (4.8-7.4)
Every characteristics^f^	NA	7.9 (5.2-10.7)

Abbreviation: NA = Not applicable.

^a^Seroprevalence adjusted according to the time of sampling, biological sex at birth, age, neighborhood.

^b^Seroprevalence adjusted according to time of dried blood spot specimen collection.

^c^Seroprevalence adjusted according to essential worker in household, parent’s ethnic minority status.

^d^Seroprevalence adjusted according to parent’s education and ethnic minority status.

^e^Seroprevalence adjusted according to household income and parent’s ethnic minority status.

^f^All of the above characteristics of the table.

In contrast, with raking, it was possible to weight the 7 identified characteristics both individually and simultaneously. In comparison with the unweighted estimate of 5.8% (95% CI,4.7-7.0), weighted estimates with raking varied from 5.3% (95% CI; 4.0, 6.6) to 7.9% (95% CI, 5.2-10.7) (Table 4, Figure 2), weighting for parental education produced the lowest estimate and weighting for all characteristics produced the highest estimate. Individual weighting by household income and parent’s ethnic/racial minority status or place of birth increased the estimated seroprevalence by 1.2, 1.7, and 0.9 percentage points, respectively. Advantages and disadvantages of both weighting methods were compared (Table 5).

**Table 5:** Advantages and disadvantages of both weighting methods.

	**Advantage**	**Disadvantage**
Marginal standardization	Produces adjusted weighted estimates that take into account variables dependencies through regression coefficients	Requires knowledge about the confounding and mediating mechanisms related to the outcome in order to estimate less-biased coefficients Requires enough data to produce stable and precise regression parameters Difficult to weight for >1 variable at the same time
Raking	Flexible and easy to implement Can produce estimates weighted for multiple variables simultaneously Only requires marginal totals or percentage points in the target population	Presume independence between variables when applying weights Algorithm convergence difficulties when data show complex patterns Weighting for multiple variables at the same time decreases result precision

Finally, sensitivity analysis between imputed and complete cases data showed overall consistency for marginal standardization estimates and for raking weighted seroprevalence. However, for both marginal standardization and the raking procedure, seroprevalence was consistently higher in weighted estimates produced from the complete case data (Appendix S1, Table S2).

## Discussion

Our results demonstrate the importance of considering weighting when a prevalence estimate based on a convenience sample is applied to a target population. With the various methods and scenarios investigated, we estimated up to a 2.1 percentage point difference between sample prevalence and target population prevalence. When the disease prevalence is rare (ie, < 10%), a 2-percentage point divergence can mean a notable difference—a 36% underestimation in our case—which can have important implications, including the potential to misdirect public health practice.^18^

There is no standard approach to verify if a sample or study population is representative of the target population. Moreover, representativeness can take on several definitions, corresponding to the nature of a random sample or simply to the idea that the sample distribution of certain characteristics is close enough to the distribution of these same characteristics in the target population.^43^ To validate representativeness under this second definition, comparison between both populations can be achieved using census data simply through investigating the differences in proportions of a given set of characteristics or through significance testing.^15^

Departures from representativity in characteristics that substantially influence the study outcome have greater potential to limit the generalizability or application of the results outside of the study population. The main strength of marginal standardization is that it uses estimated marginal effects to predict the distribution of an outcome based on the values of covariates. This produces a standardized estimate that acknowledges the dependency between multiple key factors by drawing from the strengths of statistical modeling. However, to minimize the distortion of the estimated association for each weighting covariate, a minimally sufficient adjustment set is needed to ensure the estimation of an unconfounded association without including any colliders or mediators. To identify this minimally sufficient adjustment set, scientific knowledge is needed that may not be possible for emerging pathogens and other novel diseases. Also, it was not possible in this specific study, according to our DAG, to weight for multiple factors simultaneously, and marginal standardization requires a sufficiently large sample size to be able to produce stable and precise regressions estimates. Furthermore, weighting for multiple factors simultaneously would require a minimally sufficient adjustment set that is adequate for all the weighted factors. In our application, this could not be done without adjusting for a collider or a mediator of another factor and thereby potentially introducing bias into our estimates.

The main advantage of raking is that it is easy to implement and highly flexible.^36^ Importantly, it can weight for multiple characteristics simultaneously and, therefore, give a weighted estimate that reflects a target population’s multidimensionality.^44^ A drawback of raking is that the weights are calculated as though all covariates are independent, given that the marginal totals for each characteristic are only required vs the cross-tabulated counts. This assumption of independence may not hold if the differences between the sample and the target did not occur independently in the joint distribution of some characteristics or if the characteristics are highly correlated.^23^^,^^45^ In the event of convergence issues of the raking algorithm due to complex pattern in the data, variables can be collapsed into fewer categories, but this solution make the weighting process less accurate.^23^ Weighting for multiple characteristics, as permitted by raking, is a major benefit but should be done carefully because this increases the SD and therefore decreases the precision of the estimate. Many SARS-CoV-2 seroprevalence studies implemented raking to correct their estimates, making this method more popular than marginal standardization among the current body of literature.^24-26^

### Weighting to achieve better generalizability

We found that our study population was from households that were less ethnically diverse, more educated, and wealthier compared with the target population (ie, the pediatric population of Montreal). These social characteristics have been identified as contributing to the risk of SARS-CoV-2 exposure and infection in children across multiple studies.^7^^,^^9^^,^^13^^,^^46^ Representativeness should be evaluated based on the distribution of relevant characteristics that are believed to be linked to the outcome of interest and could influence the results, based on prior scientific evidence.^47^ Two other studies found increases of 0.6% (from 3.0% to 3.6%) and 13.8% (from 2.5% to 16.3%) after weighting seroprevalence by racial/ethnic minority status.^9^^,^^21^ Considering that many seroprevalence studies reported a lack of representativity in their study population and the potential threat to generalizability,^7^^,^^13^^,^^48-50^ the differences detected between unweighted and weighted estimates in our study highlight the importance of weighting for seroprevalence estimates. Among the barriers to representativeness, online self-selection recruitment could contribute to undermining sample representativity.^15^ In-home finger prick testing, in contrast with clinic-based testing, has not been identified as an element that is susceptible to erode study representativity.^51^

Furthermore, lack of representativity might not only affect overall seroprevalence estimates, as presented here, but could also have further downstream implications, such as underestimating measures of health disparities. Indeed, weighted characteristics for our seroprevalence estimate constitute indicators of social vulnerability and weighting procedures could unveil the impact of nonrepresentativity on other measures that capture social inequalities in health, such as risk differences, risk ratios, or prevalence ratios.^52^^,^^53^

### Limitations

The first limitation of the present study is that there was likely selection bias in the cohort study, which could have led participants to differ from nonparticipants in the joint distribution of certain key characteristics that were investigated and in their risk of the outcome of interest, distorting the prevalence estimation in an unknown direction.^54^ For weighting techniques to work properly, we needed to assume that seroprevalence in every observed subgroup of our sample matched the seroprevalence in the same target population subgroups. Samples based on self-selection are prone to gather participants that differ from the target population.^55^ Random sampling should be favored over nonprobabilistic sampling to reduce selection bias, even if this could represent an issue when researchers randomly draw their sample from a clear sampling frame.^56^

Confounding bias could be present as residual confounding through the broad categorization of certain confounders or as unmeasured confounding. To reduce the influence of unmeasured confounding, we searched the literature, constructed DAGs, and identified minimally sufficient adjustment sets. The self-reporting nature of the questionnaire could have led to information bias and, more specifically, measurement error in certain factors such as household income or the presence of an essential worker in the household. However, these measurement errors are unlikely to have been differential between seropositive and seronegative children and should have little impact on our results. Also, our study did not correct for imperfect assay sensitivity, another potential source of information bias. However, a validation study indicated a 95% sensitivity and a 100% specificity of our enzyme-linked immunosorbent assay, which considerably limits the impact of false-positive and false-negative results on our estimate.^27^ Ideally, correcting for test uncertainty should be considered by seroprevalence studies to produce consistent estimates, however, the simultaneous application of multiple standardization procedures can prove challenging.^18^

Another important source of information bias was the missing data (≤30% for certain variables, such as parent’s place of birth and household income), due to the collection of certain data after the baseline questionnaire. We used MICE, assuming the hypothesis of a missing-at-random pattern in our data based on MICE’s high performance in comparison with other traditional missing data techniques, particularly when multiple auxiliary variables are integrated into the imputation model.^40^^,^^57^ Our sensitivity analysis suggests that the complete case analysis with marginal standardization would have led to an overestimation of the associations, increasing the strength of either positive or negative associations. For the complete case analysis with raking, there was an overestimation of the outcome overall weighted distribution. These results support the use of multiple imputation vs complete-case analysis, because the latter may potentially lead to more biased results.^57^

### Conclusion

This analysis highlights the limited generalizability of nonprobability sampling with unweighted estimates. For prevalence studies, the representativeness of the study population is crucial for ensuring the external validity of the results, and researchers should strive to recruit participants using probability sampling methods whenever possible. However, when random selection is not possible, to ensure generalizability of prevalence estimates, the sample’s representativity of the target population should be assessed using descriptive statistics and high-quality external data. Importantly, the representativity and generalizability of health and health inequity measures should be examined and potentially weighted if deemed necessary. Researchers interested in marginal standardization should consider increased sample sizes to allow robustness of the estimates and to meet the positivity assumption in their regression analysis. In our study, we preferred raking because of its simplicity and its capacity to simultaneously weight for multiple variables, although both approaches performed quite similarly.

## Supplementary Material

Web_Material_kwae276

## Data Availability

The data used in this study are not publicly available. Some data were copied with the authorization of the Company Canada Post. The codes used in the analysis are available at https://github.com/adriensaucier/SARS_CoV2_prevalence_weighting.
