# Supplementary material for: Generalizability of anti–SARS-CoV-2 seroprevalence estimates to the Montréal pediatric population: a comparison between 2 weighting methods
Source: Am J Epidemiol. 2024 Aug 12;194(4):1112–21. doi: 10.1093/aje/kwae276 (PMC11978615; doi:10.1093/aje/kwae276)
Supplement: Web_Material_kwae276 [file web_material_kwae276.zip › Supplementary_materials.docx]

**Supplementary Material**

**Generalizability of anti-SARS-CoV-2 seroprevalence estimates to the Montréal pediatric population: a comparison between two weighting methods**

Adrien Saucier^1^, Bouchra Nasri^1^, Britt McKinnon^1^, Mabel Carabali^1,2^, Laura Pierce^1^, Katia Charland^1^, Kate Zinszer^1^

^1^ Centre de recherche en santé publique de l’Université de Montréal, Université de Montréal, Montréal, Québec, Canada

^2^ Departement of Epidemiology, Biostatistics & Occupational Health, McGill University, Montréal, Québec, Canada

Contents

[Appendix S1 Multiple imputation by multivariate imputation by chained equations(MICE) 2](#_Toc167789284)

[Predictor selection and number of imputed datasets 2](#_Toc167789285)

[Imputation variability 3](#_Toc167789286)

[Figure S1 Variability of seroprevalence estimate across the 20 imputed datasets for marginal standardization 3](#_Toc167789287)

[Figure S2 Variability of seroprevalence estimate across the 20 imputed datasets for raking 4](#_Toc167789288)

[Variance and standard error descriptive statistics 4](#_Toc167789289)

[Table S1 Variance and standard error comparison between marginal standardization and raking 5](#_Toc167789290)

[Sensitivity analysis of imputed data versus complete cases data 5](#_Toc167789291)

[Table S2 Comparison between estimates from imputed and complete cases data estimates for marginal standardization and raking 6](#_Toc167789292)

[Appendix S2 Confounding 7](#_Toc167789293)

[Directed acyclic graph 7](#_Toc167789294)

[Figure S3 Directed acyclic graph 8](#_Toc167789295)

[Minimally sufficient adjustment sets and unbiased coefficients 9](#_Toc167789296)

[Table S3 Minimally sufficient adjustment sets for the weighted variables 9](#_Toc167789297)

[Table S4 Pooled odds ratio estimates for marginal standardization 9](#_Toc167789298)

[Spatial variability of the study population 10](#_Toc167789299)

[Table S5 Range of dispersion parameter of the marginal standardization models over the 20 imputed datasets 11](#_Toc167789300)

[Table S6 BIC comparison for models with a random effect for neighborhood 11](#_Toc167789301)

[Appendix S3 Precision of post-stratification raking and marginal standardization estimates 12](#_Toc167789302)

# Appendix S1 Multiple imputation by multivariate imputation by chained equations(MICE)

## Predictor selection and number of imputed datasets

Missing Not at Random (MNAR) patterns of missingness may be possible and to mitigate its potential effects on our results, we included a range of auxiliary participant, household, and neighborhood variables, in addition to the covariates used in our regressions, in the imputation process ^1^. These variables had little to no missingness. In our case, predictors that were integrated in the imputation process were: serological status of the child, neighbourhood of the education institution, sex assigned at birth, date of DBS, age group (2-4, 5-9, 10-14 and 15-17 years), household density (< 2 persons / bedroom, > 2 persons per bedroom), essential worker in the household, member in the household with a medical chronic condition, child having a physician, child was born premature, number of bathrooms and number of bedrooms in the household, historic PCR test positivity, median income by forward sortation area in the 2016 census, proportion of persons in the forward sortation area that were immigrants or non-permanent resident in the 2016 census. Household income before taxes and parent place of birth were not included as predictors as they contained a considerable amount of missing values (>30%). All of the variables involved in the analysis were imputed. Household ID was set as a clustering factor so siblings would more likely have similar imputed values. Imputation was made over 20 datasets (m=20 in the mice function of the mice package in R), which is aligned with the recommendations for data with low to medium missingness to ensure robustness of results^2^. Rubin’s pooling rule was applied to combine the estimates from the different datasets^2^.

## Imputation variability

The variability of the seroprevalence estimate across the 20 imputed datasets is presented in Figure S1 and Figure S2. The seroprevalence point estimates varied little across the datasets (< 0.3%), though greater variation is seen for the covariates with a larger percentage of missingness, i.e. household income and parent place of birth. Point estimates and confidence intervals across the 20 imputed datasets exhibited similar patterns for raking (S1 Figure 2). Estimates weighted for household income, parent’s place of birth and parent’s ethnic/racial minority status varied a little more than the others across the imputed datasets with the largest differences between estimates being 0.5%, 0.4% and 0.1% respectively. The largest difference between seroprevalence point estimates was 0.9%, which occurred when the estimate was weighted for all the characteristics simultaneously through raking.

### Figure S1 Variability of seroprevalence estimate across the 20 imputed datasets for marginal standardization

**
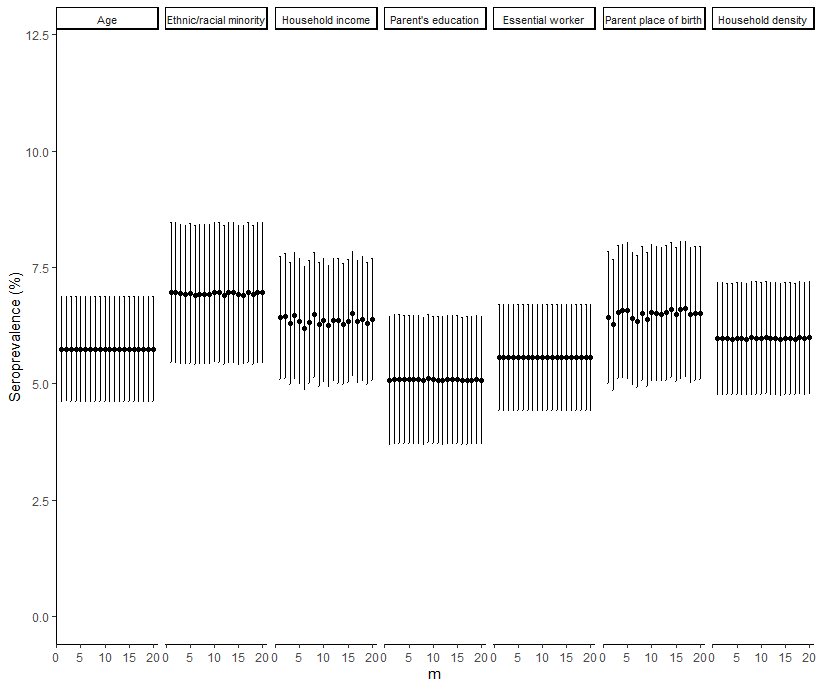
**

### Figure S2 Variability of seroprevalence estimate across the 20 imputed datasets for raking

**
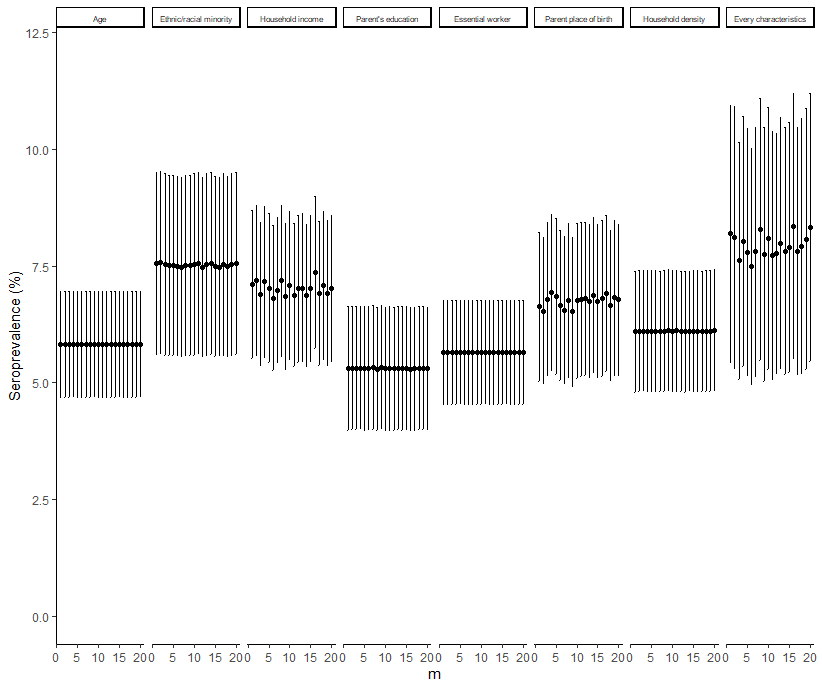
**

## Variance and standard error descriptive statistics

The variability can be separated into two main components: the within variance and the in-between variance. Within variance is the variability of the estimate within each dataset, much like the conventional definition of variance, whereas in-between variance is the additional variance caused by missingness in the data, the variance between each dataset^2^. The pooled variance consists in a sum of these two elements plus “an extra simulation variance caused by the fact that [the total variance] is estimated for finite *m*” and that correspond mathematically to the in-between variance divided by *m*^2(pt2.3)^. The in-between is therefore an indicator of how the imputation contributes to the variability of the estimate. Additionally, the proportion of the variance that is due to missingness, i.e.: the sum of the in-between and the extra simulation variance, is an informative measure to understand the impact of missingness on our results^2^. A percentage of more than 50% would signify that the impact of the imputation on the variance is larger than the complete data^2^. In Table S1, the measures are presented as standard errors, at the scale of the response variable (percentage of seroprevalence). It demonstrates that missingness contributes very little to the variability of our estimates.

### Table S1 Variance and standard error comparison between marginal standardization and raking

| Variable | Marginal standardization | | | | Raking | | | |
| --- | --- | --- | --- | --- | --- | --- | --- | --- |
|  | Average within standard error of the seroprevalence estimate | In-between standard error of the seroprevalence estimate | % of total variance due to missingness | Pooled standard error of the seroprevalence estimate | Average within standard error of the seroprevalence estimate | In-between standard error of the seroprevalence estimate | % of total variance due to missingness | Pooled standard error of the seroprevalence estimate |
|  | n=1,632 ; m=20 | | | | n=1,632 ; m=20 | | | |
| Unweighted ^a^ | 0.6 | 0.0* | 0.0* | 0.6 | 0.6 | 0.0* | 0.0* | 0.6 |
| Weighted for age ^b^ | 0.6 | 0.0* | 0.0* | 0.6 | 0.6 | 0.0* | 0.0* | 0.6 |
| Weighted for parent’s ethnic/racial minority status ^b^ | 0.8 | <0.1 | 0.1 | 0.8 | 1.0 | <0.1 | 0.1 | 1.0 |
| Weighted for household income ^c^ | 0.7 | 0.1 | 1.5 | 0.7 | 0.8 | 0.1 | 3.0 | 0.8 |
| Weighted for parent’s education ^b^ | 0.7 | <0.1 | <0.1 | 0.7 | 0.7 | <0.1 | <0.1 | 0.7 |
| Weighted for essential worker in the household ^d^ | 0.6 | <0.1 | <0.1 | 0.6 | 0.6 | <0.1 | <0.1 | 0.6 |
| Weighted for parent’s place of birth ^b^ | 0.7 | 0.1 | 1.6 | 0.7 | 0.8 | 0.1 | 2.2 | 0.8 |
| Weighted for household density ^e^ | 0.6 | <0.1 | <0.1 | 0.6 | 0.7 | <0.1 | <0.1 | 0.7 |
| Every characteristcs | NA | | NA | | 1.4 | 0.2 | 3.0 | 1.4 |

*No variable with missingness was involved in the estimate

^a^Adjusted for the time of sampling, biological sex at birth, age, neighborhood

^b^Adjusted for time of DBS

^c^Adjusted for essential worker in household, parent’s ethnic minority status

^d^Adjusted for parent’s education and ethnic minority status

^e^Adjusted for household income and parent’s ethnic minority status

## Sensitivity analysis of imputed data versus complete cases data

A final sensitivity analysis can help us understand how missingness affected our results, by comparing the estimates made from the imputed datasets and the estimates made from the complete case data (Table S2). Complete case estimates appeared to be higher than imputed data estimates. This is because that most children for which household income, parent place of birth, or any other variable was missing (n=988), they were predominantly seronegative, which reduced the denominator in a disproportionate manner in comparison to the numerator on which the seroprevalence was estimated. Using a 40% smaller complete case sample of 988 children would potentially introduce more variability and also bias than the imputed data sets. Although unweighted estimates begin higher in prevalence with the complete case data (8.0% ; 95% CI, 6.3, 9.7), compared to the imputed data (5.8% ; 95% CI 4.7, 6.9), the absolute difference between each weighting scenarios and their reference unweighted estimate appears to be consistent in that, complete case data estimates tended to produce slightly higher differences. Furthermore, the extent of the 95% confidence interval is wider among the complete case data estimates, mainly due to the smaller sample size in complete case data set and, inversely, to the fact that imputation, as seen in part 3 of this appendix, does not add much uncertainty to our estimates.

### Table S2 Comparison between estimates from imputed and complete cases data estimates for marginal standardization and raking

| Variable | Marginal standardization | | | | | | Raking | | | | | |
| --- | --- | --- | --- | --- | --- | --- | --- | --- | --- | --- | --- | --- |
|  | Pooled seroprevalence in imputed data  (95% CI) | Margin of error in imputed data | Absolute difference with unweighted estimate | Seroprevalence in complete cases data  (95% CI) | Margin of error  in complete cases data | Absolute difference with unweighted estimate | Seroprevalence in imputed data  (95% CI) | Margin of error in imputed data | Absolute difference with unweighted estimate | Seroprevalence in complete cases data  (95% CI) | Margin of error  in complete cases data | Absolute difference with unweighted estimate |
|  | n=1,632 ; m=20 | | | n=988 | | | n=1,632 ; m=20 | | | n=988 | | |
| Unweighted | 5.8  (4.7, 6.9) *^a^* | ±1.1 | NA | 8.0  (6.3, 9.7) *^a^* | ±1.7 | NA | 5.8  (4.7, 7.0) | ±1.2 | NA | 8.0  (6.3, 9.7) | ±1.7 | NA |
| Weighted for age | 5.8  (3.9, 7.7) *^b^* | ±1.2 | 0.0 | 8.1  (6.3, 9.8) *^b^* | ±1.7 | 0.1 | 5.8  (4.7, 7.0) | ±1.2 | 0.0 | 8.1  (6.4, 9.9) | ±1.8 | 0.1 |
| Weighted for parent’s ethnic/racial minority status | 6.9  (5.4, 8.4) *^b^* | ±1.5 | 1.1 | 9.6  (7.4, 11.8) *^b^* | ±2.2 | 1.6 | 7.5  (5.6, 9.5) | ±2.0 | 1.7 | 10.5  (7.6, 13.4) | ±2.9 | 2.5 |
| Weighted for household income | 6.3  (5.0, 7.7) *^c^* | ±1.4 | 0.5 | 9.1  (7.0, 11.1) *^c^* | ±2.0 | 1.1 | 7.0  (5.4, 8.6) | ±1.6 | 1.2 | 10.1  (7.7, 12.6) | ±2.5 | 2.1 |
| Weighted for parent’s education | 5.1  (3.7, 6.5) *^b^* | ±1.4 | 0.7 | 6.8  (4.5, 9.1) *^b^* | ±2.3 | 1.2 | 5.3  (4.0, 6.6) | ±1.3 | 0.5 | 7.1  (5.0, 9.2) | ±2.1 | 0.9 |
| Weighted for essential worker in the household | 5.6  (4.4, 6.7) *^d^* | ±1.1 | 0.2 | 7.6  (5.9, 9.3) *^d^* | ±1.7 | 0.4 | 5.7  (4.5, 6.8) | ±1.1 | 0.1 | 7.7  (6.1, 9.4) | ±1.7 | 0.3 |
| Weighted for parent’s place of birth | 6.5  (5.0, 7.9) *^b^* | ±1.5 | 0.7 | 9.3  (7.1, 11.5) *^b^* | ±2.2 | 1.3 | 6.7  (5.1, 8.4) | ±1.7 | 0.9 | 9.8  (7.2, 12.4) | ±2.6 | 1.8 |
| Weighted for household density | 6.0  (4.8, 7.2) *^e^* | ±1.3 | 0.2 | 8.2  (6.4, 9.9) *^e^* | ±1.7 | 0.2 | 6.1  (4.8, 7.4) | ±1.3 | 0.3 | 8.3  (6.4, 10.1) | ±1.8 | 0.3 |
| Every characteristcs | NA | | | NA | | | 7.9  (5.2, 10.7) | ±2.8 | 2.1 | 11.7  (7.2, 16.3) | ±4.6 | 3.7 |

^a^Seroprevalence adjusted according to the time of sampling, biological sex at birth, age, neighborhood

^b^Seroprevalence adjusted according to time of DBS

^c^Seroprevalence adjusted according to essential worker in household, parent’s ethnic minority status

^d^Seroprevalence adjusted according to parent’s education and ethnic minority status

^e^Seroprevalence adjusted according to household income and parent’s ethnic minority status

# Appendix S2 Confounding

This appendix describes the steps taken to develop a directed acyclic graph (DAG) and to diminish the impact of confounding on our marginal standardization weighting results and how spatial variability from the study population was managed.

## Directed acyclic graph

A directed acyclic graph (DAG) was constructed based on the literature on SARS-CoV-2 infection risk factors among children and adolescents, which was used to guide the selection of the variables to be included in our marginal standardization models (Figure S3). More precisely, the DAG allowed us to built parsimonious models by identifying the minimally sufficient adjustment set that is required to minimize the impact of confounding on our regression coefficients.

The main risk factors that were identified as being associated with our outcome included race and ethnicity, socio-economical status, living with a healthcare worker and household overcrowding. Pediatric seroprevalence studies found an association between belonging to an ethnic minority or racial group and the probability of seropositivity ^3,4^. Globally, socioeconomic status has been systematically associated with risk of SARS-CoV-2 infection, at the ecological or individual level ^5–8^. Literature on the association between socioeconomic status and SARS-CoV-2 infection in pediatric populations specifically, is tenuous. However, there is evidence that this association is present in younger age groups ^9^. We used « parental education » and « household income » as proxies to capture child socio-economic status. Furthermore, it has been shown that living with a healthcare worker increases the probability of a SARS-CoV-2 infection among children^4,10^. Finally, overcrowding was identified as an risk factor for infection in children^3,11^.

### Figure S3 Directed acyclic graph

**
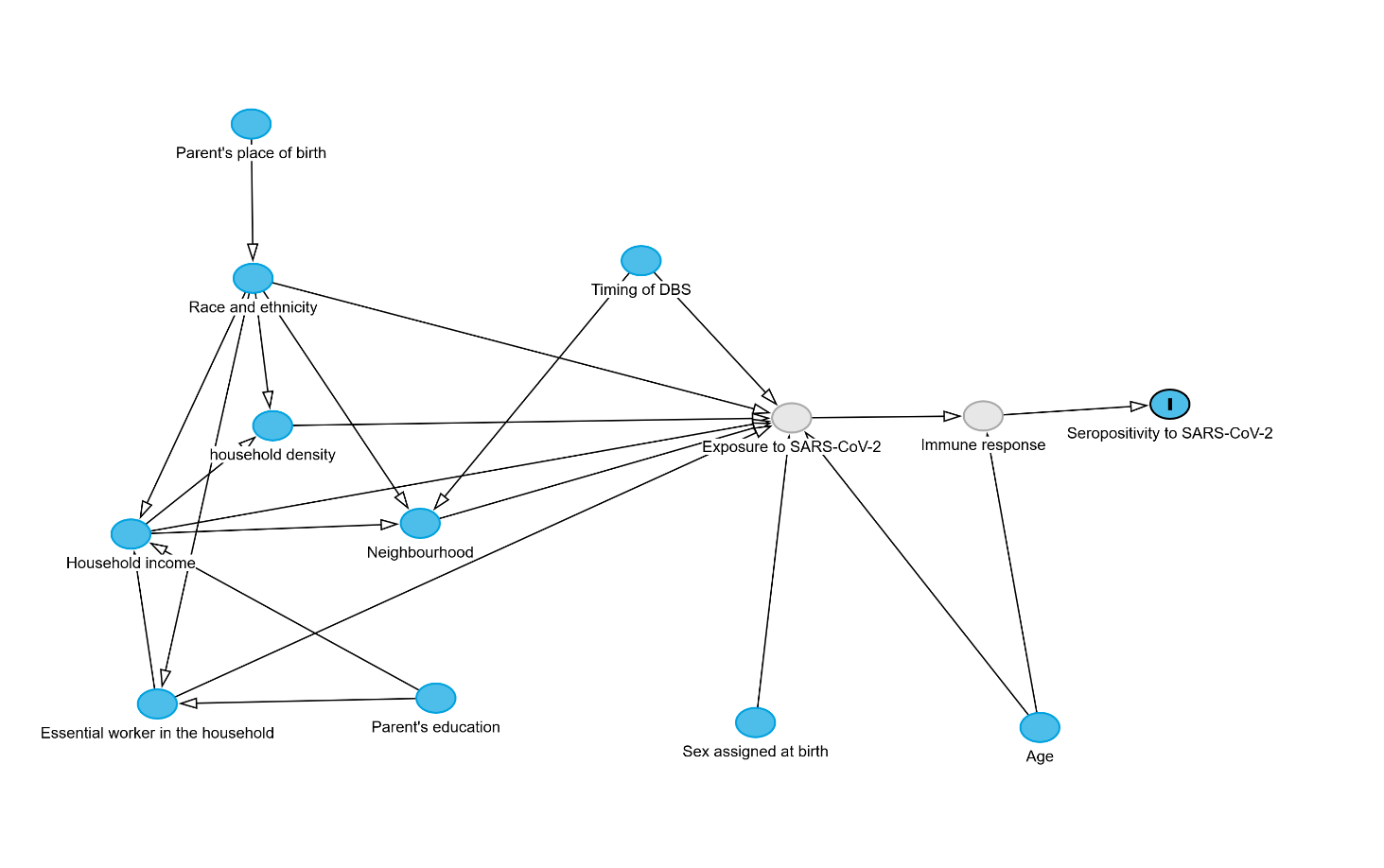
**

## Minimally sufficient adjustment sets and unbiased coefficients

The minimally sufficient adjustment set was identified for each weighted factor individually, as seen in Table S3. As it can be deduced from this table and Figure S3, it was impossible to identify a set of adjustments that would diminish confounding for all factors simultaneously, because one or more confounders for one weighting variable was a collider or mediator for another weighting variable. By including colliders/mediators we would introduce confounding. Indeed, since marginal standardization requires we use unbiased regression coefficients in the weighting process, a more conservative and parsimonious approach led us to suggest plausible scenarios that weighted for only one factor at the time. The “marginal effects” used in our analysis are presented in the Table S4.

### Table S3 Minimally sufficient adjustment sets for the weighted variables

| Weighted covariate | Minimally sufficient adjustment set | Additional adjustments |
| --- | --- | --- |
| Age |  | Timing of DBS |
| Parent’s ethnic minority status |  | Timing of DBS |
| Household income | Essential worker in household, Parent’s ethnic minority status | Timing of DBS |
| Parent’s education |  | Timing of DBS |
| Essential worker in the household | Parent’s education, Parent’s ethnic minority status |  |
| Parent’s place of birth |  | Timing of DBS |
| Household density | Household income, Parent’s ethnic minority status |  |

### Table S4 Pooled odds ratio estimates for marginal standardization

|  | Basic model^a^  OR  (95% CI) | Model for age weighting^b^  OR  (95% CI) | Model for ethnic minority weighting^b^  OR  (95% CI) | Model for household income weighting^c^  OR  (95% CI) | Model for education weighting^b^  OR  (95% CI) | Model for essential worker in household weighting^d^  OR  (95% CI) | Model for parent’s place of birth weighting^b^  OR  (95% CI) | Model for household density weighting^e^  OR  (95% CI) |
| --- | --- | --- | --- | --- | --- | --- | --- | --- |
| n=1,632 | | | | | | | | |
| Timing of DBS | | | | | | | | |
| Oct-Nov 2020 | Reference | Reference | Reference | Reference | Reference |  | Reference |  |
| Dec-Jan 2021 | 1.54  (0.67, 3.51) | 1.81  (0.91, 3.58) | 1.81  (0.91, 3.61) | 1.51  (0.76, 3.02) | 1.74  (0.88, 3.73) |  | 1.65  (0.83, 3.28) |  |
| Feb-Apr 2021 | 2.89  (1.17, 7.16)* | 3.80  (1.93, 7.49) | 3.67  (1.86, 7.25)* | 3.03  (1.53, 6.03)* | 3.71  (1.89, 7.29)* |  | 3.40  (1.73, 6.68)* |  |
| Biological sex at birth | | | | | | | | |
| Male | Reference |  |  |  |  |  |  |  |
| Female | 1.44  (0.60, 2.98) |  |  |  |  |  |  |  |
| Age | | | | | | | | |
| 2-4 | Reference | Reference |  |  |  |  |  |  |
| 5-9 | 1.16  (0.60, 2.22) | 1.24  (0.65, 2.35) |  |  |  |  |  |  |
| 10-14 | 1.61  (0.86, 3.02) | 1.73  (0.94, 3.20) |  |  |  |  |  |  |
| 15-17 | 1.59  (0.76, 3.33) | 1.76  (0.86, 3.62) |  |  |  |  |  |  |
| Neighbourhood | | | | | | | | |
| West Island | Reference |  |  |  |  |  |  |  |
| Plateau-Mont-Royal | 1.15  (0.56, 2.39) |  |  |  |  |  |  |  |
| Mercier-Hochelaga-Maisonneuve | 1.33  (0.60, 2.98) |  |  |  |  |  |  |  |
| Montréal-Nord | 1.58  (0.70, 3.61) |  |  |  |  |  |  |  |
| Parent’s ethnic minority status | | | | | | | | |
| No |  |  | Reference | Reference |  | Reference |  | Reference |
| Yes |  |  | 2.09  (1.26, 3.47)* | 1.92  (1.14, 3.23)* |  | 2.25  (1.36, 3.73)* |  | 1.94  (1.16, 3.25)* |
| Household density | | | | | | | | |
| < 2 persons / bedroom |  |  |  |  |  |  |  | Reference |
| > 2 persons per bedroom |  |  |  |  |  |  |  | 1.26  (0.73, 2.20) |
| Parent’s education | | | | | | | | |
| Less than bachelor degree |  |  |  |  | Reference | Reference |  |  |
| Bachelor degree |  |  |  |  | 1.58  (0.89, 2.81) | 1.44*  (0.81, 2.56) |  |  |
| Master degree or more |  |  |  |  | 1.47  (0.82, 2.65) | 1.41  (0.78, 2.52) |  |  |
| Essential worker in household | | | | | | | | |
| No essential worker |  |  |  | Reference |  | Reference |  |  |
| At least one essential worker, health domain |  |  |  | 1.56  (0.90, 2.69) |  | 1.64  (0.95, 2.82) |  |  |
| At least one essential worker, not health |  |  |  | 1.50  (0.92, 2.44) |  | 1.49  (0.92, 2.40) |  |  |
| Household income | | | | | | | | |
| <100,000$ |  |  |  | Reference |  |  |  | Reference |
| >100,000$ |  |  |  | 0.67  (0.42, 1.05) |  |  |  | 0.57  (0.37, 0.90)* |
| Parent’s place of birth | | | | | | | | |
| In Canada |  |  |  |  |  |  | Reference |  |
| Outside Canada |  |  |  |  |  |  | 1.48  (0.94, 2.35) |  |

^a^Adjusted according to the time of DBS, biological sex at birth, age, neighborhood

^b^Adjusted according to time of DBS

^c^Adjusted according to essential worker in household, parent’s ethnic minority status

^d^Adjusted according to parent’s education and ethnic minority status

^e^Adjusted according to household income and parent’s ethnic minority status

## Spatial variability of the study population

It was assumed that relevant neighborhood characteristics associated with the outcome were captured, as depicted in the DAG. Neighborhood of residency is a broad factor that could encompass many characteristics, some of them potentially being colliders or mediators between the weighted factor and our outcome. For this particular reason, we preferred to adjust only for the minimally sufficient adjustment sets in order to control only for factors that we know to be confounders of the relation between the weighted factor and the outcome. To support this assumption, we assessed the results of a quasi-binomial model to determine whether the dispersion parameter was larger than 1, indicating overdispersion. All models reported a dispersion parameter of approximately 1.00 (Table S5), which suggest that no significant clustering patterns were present in the data. In addition, we evaluated if the addition of a random effect for the neighborhood of the education institution in our models would improve the fit with the data, using the Bayesian information criteria (BIC) as a measure of fit (Table S6). Models with the random effect for neighborhood did not perform better than the models we used in our marginal standardization weighting (without random effect for neighborhood), which supports the assumption of a lack of neighborhood clustering of the data.

### Table S5 Range of dispersion parameter of the marginal standardization models over the 20 imputed datasets

| Models | Range of dispersion parameter |
| --- | --- |
|  | n=1,632; m=20 |
| Unweighted model ^a^ | 1.00, 1.00 |
| Age model ^b^ | 1.00, 1.00 |
| Parent’s ethnic/racial minority status model ^b^ | 1.00, 1.01 |
| Household income model ^c^ | 1.00, 1.01 |
| Parent’s education model ^b^ | 0.99, 0.99 |
| Essential worker in the household model ^d^ | 1.00, 1.00 |
| Parent’s place of birth model ^b^ | 1.00, 1.01 |
| Household density model ^e^ | 1.00, 1.00 |
| Saturated model^f^ | 0.99, 1.02 |

^a^Adjusted for the time of DBS biological sex at birth, age

^b^Adjusted for time of DBS,

^c^Adjusted for essential worker in household, parent’s ethnic minority status

^d^Adjusted for parent’s education and ethnic minority status

^e^Adjusted for household income and parent’s ethnic minority status

^f^Adjusted for the time of DBS, biological sex at birth, age, essential worker in household, parent’s ethnic minority status, household income, parent’s place of birth and parent’s education

### Table S6 BIC comparison for models with a random effect for neighborhood

| Models | Minimally sufficient adjustment model | Random effect model for neighborhood |
| --- | --- | --- |
|  | n=1,632; m=20 | |
| Unweighted model ^a^ | 750.27 | 757.67 |
| Age model ^b^ | 745.79 | 753.19 |
| Parent’s ethnic/racial minority status model ^b^ | 728.09 | 735.49 |
| Household income model ^c^ | 742.73 | 750.13 |
| Parent’s education model ^b^ | 740.04 | 747.44 |
| Essential worker in the household model ^d^ | 754.48 | 758.52 |
| Parent’s place of birth model ^b^ | 732.49 | 739.89 |
| Household density model ^e^ | 738.34 | 745.00 |

^a^Adjusted for the time of DBS, age and biological sex at birth

^b^Adjusted for time of DBS

^c^Adjusted for essential worker in household, parent’s ethnic minority status

^d^Adjusted for parent’s education and ethnic minority status

^e^Adjusted for household income and parent’s ethnic minority status

# Appendix S3 Precision of post-stratification raking and marginal standardization estimates

This appendix compares the precision of post-stratification raking and marginal standardization estimates and demonstrates that neither approach systematically produces more precise estimates, but that their performance depends on the context of the data.

Suppose we observe *Y_i_,X*_1_*_i_,X*_2_*_i_* ∈ {0*,*1}, where *W* is the post-stratification matrix defined by *w_jk_*, the true proportion of *X*_1_*_i_* = *j* and *X*_2_*_i_* = *k* in the population. Further set *w_j_*_·_ = *w_j_*_0_ +*w_j_*_1_ (the true proportion of *X*_1_*_i_* = *j* in the population) and *w*_·_*_k_* = *w*_0_*_k_* + *w*_1_*_k_* (the true proportion of *X*_2_*_i_* = *k* in the population).

We want to compare the full post-stratification, corresponding to a large sample from the data with probabilities *W*, with the marginal standardization, which amount to post-stratification with weights *w*_1_ for *X*_1_ and weights *w*_2_ for *X*_2_.

More precisely, if *A_jk_* = {*i*;*x*_1_*_i_* = *j,x*_2_*_i_* = *k*}, then one resamples from *A_jk_* with probability *w_jk_*. The marginal standardization (for *X*_1_) consists in resampling from *A_j_*_·_ = {*i*;*x*_1_*_i_* = *j*} with probability *w_j_*_·_.

To simplify the calculation, instead of considering the binomial logit model, we consider 2-way ANOVA, i.e., the model is

*Yi* = *β*0 + *β*1*Xi*1 + *β*2*Xi*2 + *β*12*Xi*1*Xi*2 + *ϵi, i* ∈ {1*,...,n*}*.*

For simplicity, we took *j* = *k* = 0. Then *β*_0_ is estimated by *m*_00_, where
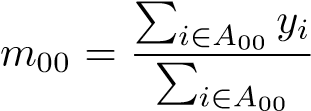
. Further

set *n*_00_ = |*A*_00_|, the cardinality of *A*_00_. Let *I_j_* be the selected state at draw *j*. *j* ∈ {1*,...,N*}. The post-stratification estimator *m*_00_*_,p_* is then given by


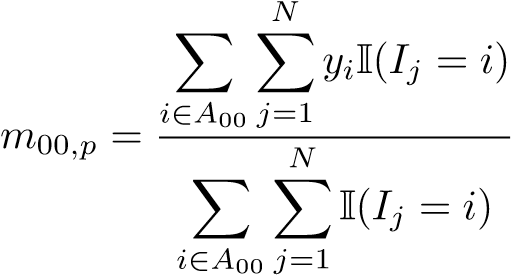
*.*

If *N* is large enough,
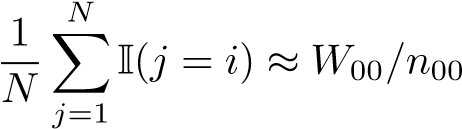
, so


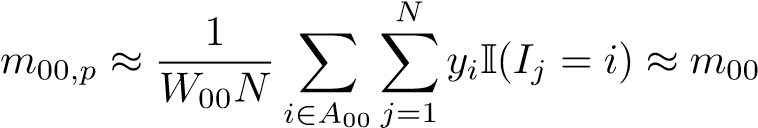
*.*

For the marginal standardisation given *X*_1_, the estimator of *β*_0_ is


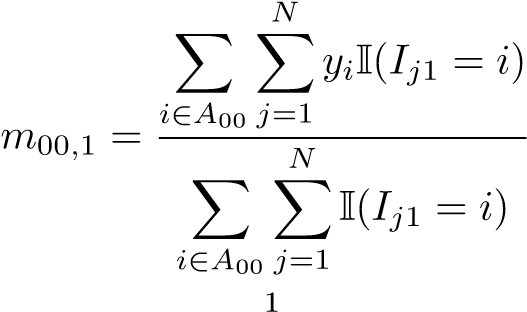
*,*

with
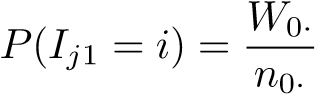
. Hence, if *N* is large enough,


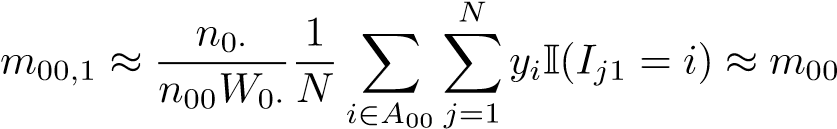
*.*

**Remark:** *We could get a better approximation by using ratio estimators, but the present approximation is good enough for illustration.*

In fact, for any resampled estimator


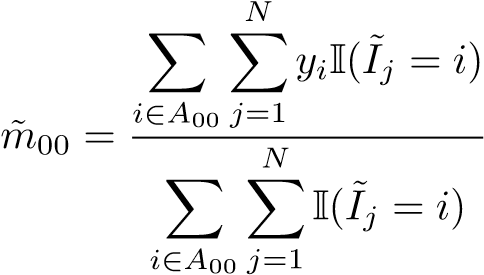
*,*

with *P*(*I*^˜^*_j_* = *i*) = *p*_0_, we have


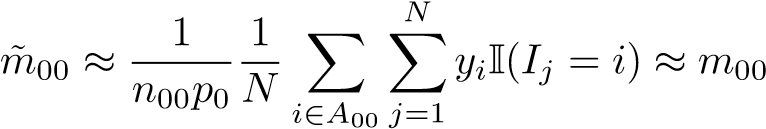
*.*

As a result, the conditional variance of *m*˜ _00_ is approximately
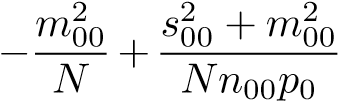
. It follows that the variance of post-stratification for *µ*_00_ = *β*_0_ is smaller than the one of marginal standardization with respect to
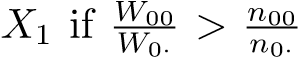
, i.e., the empirical ratio
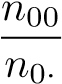
 is smaller than the theoretical ratio
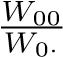
. This can be computed in advance.

Similarly, the variance of post-stratification for *µ*_00_ = *β*_0_ is smaller than the one of marginal standardization with respect to
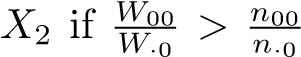
, i.e., the empirical ratio
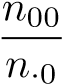
 is smaller than the theoretical ratio
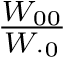
.

Note that all previous computations can be done for the estimator of *µ_jk_* = *E*(*Y* |*X*_1_ = *j,X*_2_ = *k*), by simply replacing 00 by *jk*. More precisely, the variance of post-stratification for *µ_jk_* is smaller than the one of marginal standardization with respect to
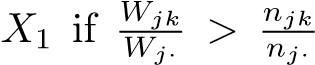
, and the variance of post-stratification for *µ_jk_* is smaller than the one of marginal standardization with respect to *X*_2_ if


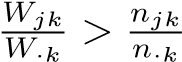
.

**Remark:** For *k* fixed,
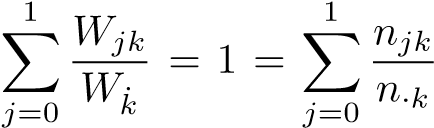
, **so the post-stratification cannot be always**

**better than the marginal standardizatio with respect to** *X*_2_ **for all** *µ_jk_***. The same reasoning holds for the marginal standardization with respect to** *X*_1_**.**

**Figure S3 Directed acyclic graph legend**

**
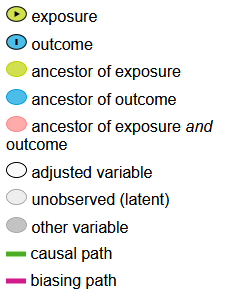
**

**References**

1. Buuren S van, Groothuis-Oudshoorn K. mice : Multivariate Imputation by Chained Equations in *R*. J Stat Soft [Internet]. 2011 [cited 2022 Nov 24];45(3). Available from: http://www.jstatsoft.org/v45/i03/

2. Stef van Buuren. Flexible Imputation of Missing Data [Internet]. CRC Press; 2018 [cited 2023 Jan 26]. (Interdisciplinary Statistics Series). Available from: https://stefvanbuuren.name/fimd/workflow.html

3. Levorson RE, Christian E, Hunter B, Sayal J, Sun J, Bruce SA, et al. A cross-sectional investigation of SARS-CoV-2 seroprevalence and associated risk factors in children and adolescents in the United States. PLoS One. 2021;16(11):e0259823.

4. Ladhani SN, Baawuah F, Beckmann J, Okike IO, Ahmad S, Garstang J, et al. SARS-CoV-2 infection and transmission in primary schools in England in June-December, 2020 (sKIDs): an active, prospective surveillance study. Lancet Child Adolesc Health. 2021 Jun;5(6):417–27.

5. Lefebvre G, Haddad S, Moncion-Groulx D, Saint-Onge M, Dontigny A. Socioeconomic disparities and concentration of the spread of the COVID-19 pandemic in the province of Quebec, Canada. BMC Public Health. 2023 Jun 6;23(1):1096.

6. Hawkins RB, Charles EJ, Mehaffey JH. Socio-economic status and COVID-19-related cases and fatalities. Public Health. 2020 Dec;189:129–34.

7. Upshaw TL, Brown C, Smith R, Perri M, Ziegler C, Pinto AD. Social determinants of COVID-19 incidence and outcomes: A rapid review. PLoS One. 2021;16(3):e0248336.

8. Karmakar M, Lantz PM, Tipirneni R. Association of Social and Demographic Factors With COVID-19 Incidence and Death Rates in the US. JAMA Network Open. 2021 Jan 29;4(1):e2036462.

9. Sahli S, Størdal K. Systematic review of socioeconomic factors and COVID-19 in children and adolescents. Acta Paediatrica [Internet]. [cited 2024 Feb 2];n/a(n/a). Available from: https://onlinelibrary.wiley.com/doi/abs/10.1111/apa.17091

10. Boey L, Roelants M, Merckx J, Hens N, Desombere I, Duysburgh E, et al. Age-dependent seroprevalence of SARS-CoV-2 antibodies in school-aged children from areas with low and high community transmission. Eur J Pediatr. 2021 Aug 28;1–8.

11. Werzberger A, Carreño JM, Team SS, Polinger A, Krammer F, Zachariah P. Household level SARS-CoV-2 sero-epidemiology in a high prevalence group of adults and children-implications for community infection control. Am J Infect Control. 2021 Nov;49(11):1438–40.
